# An atlas for hemocytes in an insect

**DOI:** 10.7554/eLife.59113

**Published:** 2020-06-30

**Authors:** Samuel Liegeois, Dominique Ferrandon

**Affiliations:** 1 Institut de Biologie Moléculaire et Cellulaire, Université de Strasbourg, UPR 9022 du CNRSStrasbourgFrance; 2 Sino-French Hoffmann Institute, Guangzhou Medical UniversityGuangzhouChina

**Keywords:** blood, hemocytes, scRNA-seq, wounding, wasp infestation, immune response, *D. melanogaster*

## Abstract

Single-cell RNA sequencing has revealed distinct subpopulations of hemocytes in fruit fly larvae.

**Related research article** Tattikota SG, Cho B, Liu Y, Hu Y, Barrera V, Steinbaugh MJ, Yoon SH, Comjean A, Li F, Dervis F, Hung RJ, Nam JW, Ho Sui S, Shim J, Perrimon N. 2020. A single-cell survey of *Drosophila* blood. *eLife* **9**:e54818. doi: 10.7554/eLife.54818

The discovery of phagocytosis – the process through which a cell can engulf a pathogen or other object – by the Russian zoologist Élie Metchnikow in 1882 ushered in the era of cellular immunity. However, progress in this field has been modest in insects, even in the genetic workhorse *Drosophila melanogaster*. The prevailing view is that hemocytes, the blood cells involved in the immune response, are divided into just three populations in fruit flies. Even though morphological studies hint at more complexity (Rizki, 1957), hemocyte subpopulations in insects remain poorly understood.

Now, in eLife, Norbert Perrimon (Harvard Medical School) and colleagues – including Sudhir Gopal Tattikota as first author, and co-workers at Harvard and Hanyang University – report a single-cell RNA sequencing study of the hemocytes of *D. melanogaster* larvae, providing a molecular 'atlas' of hemocyte gene expression (Tattikota et al., 2020).

Hematopoiesis, the process through which blood cells are made, occurs in two distinct waves during development in *D. melanogaster*. The first wave happens during embryogenesis, and the hemocytes produced during this wave proliferate further during the larval stages of development. The second wave occurs in an organ called the larval lymph gland, which releases hemocytes at the onset of metamorphosis (Banerjee et al., 2019). About 60% of the hemocytes in an adult fly were produced during the first wave, with about 40% being in the lymph glands during the second wave: hematopoiesis does not occur in adult flies (Sanchez Bosch et al., 2019). Recent work underlines the fact that cells from any of the three major populations can be derived either from the first or second wave of hematopoiesis, yet display a similar molecular signature despite their distinct origins (Cattenoz et al., 2020; Cho et al., 2020).

The three populations of hemocytes are plasmatocytes (about 95% of the total), crystal cells (which have a critical role in the process by which larvae produce melanin), and lamellocytes (which are generally only seen during pupation). Some lamellocytes are produced by the lymph glands while others derive from plasmatocytes, when a larva undergoes an ‘immune’ challenge (such as an injury or infestation by parasitoid wasps).

Tattikota et al. isolated single cells from *D. melanogaster* larvae that were either healthy or had been subject to one of two ‘immune’ challenges: some larvae had been wounded with a fine tungsten needle, and others had been infested by parasitoid wasps. Single-cell RNA sequencing was performed and the hemocytes were classified into different subpopulations according to the genes they were expressing. This analysis revealed 12 subpopulations of plasmatocytes (named PM1 to PM12), two subpopulations of crystal cells, and two subpopulations of lamellocytes (Figure 1).

**Figure 1.**
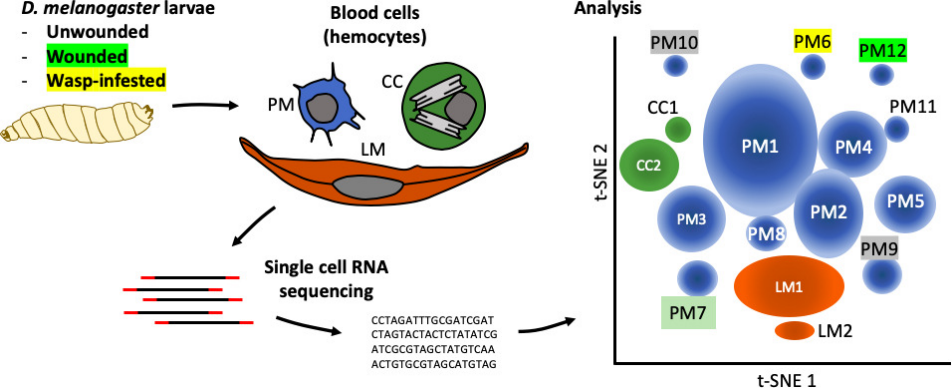
The complexity within hemocyte populations in *D. melanogaster* larvae revealed by single-cell RNA sequencing. RNA-seq analysis was performed on cells taken from *D. melanogaster* larvae that were either: (i) healthy; (ii) had been wounded with a tungsten needle; (iii) had been infested with parasitoid wasps (left). The three types of larval hemocytes – plasmatocytes (PM), crystal cells (CC) and lamellocytes (LL) – had their RNA extracted and analyzed to distinguish subpopulations within these groups (center). 12 plasmatocyte subpopulations (PM1–12), two crystal cell subpopulations (CC1, CC2), and two lamellocyte subpopulations (LM1, LM2) were defined, based on the clusters they formed when their gene expression was analyzed (right). Gene expression in these subpopulations can be accessed at www.flyrnai.org/scRNA/blood.

PM1 was the largest population, representing 30% of the cells, and it remains poorly defined in terms of specific molecular markers. For instance, the gene *NimC1*, which encodes a receptor protein that corresponds to the pan-plasmatocyte antigen marker P1 (Kurucz et al., 2007), is only expressed in between 10% and 25% of plasmatocytes. This may indicate that although *NimC1* is only expressed in plasmatocytes during differentiation, the protein it encodes remains on the surface of these cells. 10% of the cells analyzed belonged to a population of dividing plasmatocytes, called PM2, which is in keeping with the number of hemocytes increasing during larval stages. These cells likely represent the progenitors of all three mature cell type lineages.

While some of the 12 subpopulations of plasmatocytes were present in healthy larvae and in larvae that had been subject to an ‘immune’ challenge, two populations that expressed antimicrobial peptide genes mostly appeared in cells that had been subject to a challenge. All cells in the PM6 and PM7 populations expressed antimicrobial *Cecropin* genes, but the expression of receptors belonging to the corresponding immune response pathway was modest and found in no more than about 20% of these cells.

Tattikota et al. also found a subset of crystal cells that expressed the gene for a fibroblast growth factor (FGF)-like ligand called *branchless*. The gene that codes for the receptor of this ligand, *breathless*, was expressed in a subset of lamellocytes. Further tests on the larvae that had been infested by a parasitoid wasp led the researchers to conclude that FGF signaling is required for the formation of a cellular capsule around the eggs laid by the wasp. Overall, these experiments suggest that the crystal cells expressing *branchless* drive the maturation and possibly the recruitment of lamellocytes to the wasp eggs.

This work will allow future genetic studies to better understand the roles of these subpopulations in development and in *D. melanogaster* immunity. Indeed, using the *Drosophila* tool box, it will be possible to manipulate gene expression in the different subpopulations. However, further validation work will be required. For instance, subpopulations of plasmatocytes have also been discerned in larvae by another group (Cattenoz et al., 2020), yet the markers used to define some of these subpopulations are either expressed in all the cells studied by Tattikota et al., or are hardly expressed at all.

The markers identified in these studies will be useful to investigate the role of hemocyte populations in metamorphosis or in infection models (Cattenoz et al., 2020; Cho et al., 2020; Tattikota et al., 2020). Infections are more efficiently investigated at the adult stage as larvae exhibit a complex pattern of hormonal regulation, are highly sensitive to wounds, and develop into pupae within hours. Since important differences were observed in the expression pattern of hemocyte genes during embryogenesis and larval stages (Cattenoz et al., 2020), it is likely that adult hemocytes express a diverse set of genes dependent on their previous roles (Weavers et al., 2016). Single-cell RNA-seq studies will therefore also have to be performed in adult flies to provide a meaningful context for understanding the cellular immune response during infections.
